# Identification and Functional Characterization of Two Intronic* NIPBL* Mutations in Two Patients with Cornelia de Lange Syndrome

**DOI:** 10.1155/2016/8742939

**Published:** 2016-01-26

**Authors:** María E. Teresa-Rodrigo, Juliane Eckhold, Beatriz Puisac, Jelena Pozojevic, Ilaria Parenti, Carolina Baquero-Montoya, María C. Gil-Rodríguez, Diana Braunholz, Andreas Dalski, María Hernández-Marcos, Ariadna Ayerza, María L. Bernal, Feliciano J. Ramos, Dagmar Wieczorek, Gabriele Gillessen-Kaesbach, Juan Pié, Frank J. Kaiser

**Affiliations:** ^1^Unit of Clinical Genetics and Functional Genomics, Departments of Pharmacology-Physiology and Pediatrics, School of Medicine, University of Zaragoza, CIBERER-GCV and ISS-Aragon, 50009 Zaragoza, Spain; ^2^Section of Functional Genetics, Institute of Human Genetics, University of Lübeck, 23538 Lübeck, Germany; ^3^Department of Health Sciences, Medical Genetics, University of Milan, 20122 Milan, Italy; ^4^Department of Pediatrics, Pablo Tobon Uribe Hospital, 05001000 Medellín, Colombia; ^5^Institute of Human Genetics, University of Lübeck, 23538 Lübeck, Germany; ^6^Institute of Human Genetics, University Hospital Düsseldorf, Heinrich-Heine University, 40225 Düsseldorf, Germany

## Abstract

Cornelia de Lange syndrome (CdLS) is a rare genetically heterogeneous disorder with a high phenotypic variability including mental retardation, developmental delay, and limb malformations. The genetic causes in about 30% of patients with CdLS are still unknown. We report on the functional characterization of two intronic* NIPBL* mutations in two patients with CdLS that do not affect a conserved splice-donor or acceptor site. Interestingly, mRNA analyses showed aberrantly spliced transcripts missing exon 28 or 37, suggesting the loss of the branch site by the c.5329-15A>G transition and a disruption of the polypyrimidine by the c.6344del(-13)_(-8) deletion. While the loss of exon 28 retains the reading frame of the* NIBPL* transcript resulting in a shortened protein, the loss of exon 37 shifts the reading frame with the consequence of a premature stop of translation. Subsequent quantitative PCR analysis demonstrated a 30% decrease of the total* NIPBL* mRNA levels associated with the frameshift transcript. Consistent with our results, this patient shows a more severe phenotype compared to the patient with the aberrant transcript that retains its reading frame. Thus, intronic variants identified by sequencing analysis in CdLS diagnostics should carefully be examined before excluding them as nonrelevant to disease.

## 1. Introduction

Cornelia de Lange syndrome (CdLS; OMIM 1227470, 300590, 610759, 614701, and 300882) is a rare congenital disorder characterized by developmental delay, typical facial dysmorphism, limb malformations, and gastrointestinal and neurological problems [1]. CdLS is caused by mutations affecting the cohesin complex, which participates in essential cell processes such as chromosome segregation during cell division, DNA repair and replication, and gene expression [2]. Mutations have been identified in five different genes (*NIPBL*,* SMC1A*,* SMC3*,* HDAC8,* and* RAD21*), encoding structural or regulatory components of cohesin [2–9].

The* NIPBL* gene is located on chromosome 5p13.2. At least 60% of the patients with the clinical diagnosis of CdLS show* NIPBL* mutations [3–5].* NIPBL* spans more than 190 kb and contains 47 exons [10]. In addition to the previously reported two isoforms, four new shorter transcripts that exclude different combinations of exons 10, 12, 33 + 34, or 45 have been described recently [11].

Although mutations predicted to affect* NIPBL* splicing represent about 15% of all known mutations in* NIPBL*, their functional consequences on mRNA splicing are often unknown [12]. Accurate mRNA splicing requires several functional DNA elements, including a splice-donor and acceptor site, a polypyrimidine tract, a branch point, and further regulatory sequences that can either be located within intronic or exonic regions of the gene body and act as enhancers or silencers of the splicing process and are mostly unknown [13]. Mutations in the highly conserved AG/GT splice-acceptor and donor dinucleotides almost always result in aberrant splicing [14], while the pathological consequences of mutations in the other functional elements are mostly unknown. To distinguish the latter mutations from intronic polymorphisms, extensive* in vitro* analyses on mRNA extracted from patient samples are needed [15].

In this paper we report for the first time on the identification and functional investigation of two intronic* NIPBL* mutations that do not affect the conserved splice-donor or acceptor sites. Sequencing analysis of the parents could confirm the* de novo* status of either mutation in the patients. We used* in vitro* as well as* in vivo* analyses to confirm the functional consequences of both mutations, and we have tried to correlate our findings with the phenotype of the patients.

## 2. Patients and Methods

### 2.1. Patients and Controls

This study includes two German patients who meet clinical criteria for CdLS according to Kline et al. [1]. The ethical standards of the Declaration of Helsinki have been followed. Patients' parents have written informed consent to participate in the study. A pool of four cDNAs from normal individuals was used as a control to perform the experiments.

### 2.2. DNA Extraction and Sequence Analyses

Genomic DNA was extracted from peripheral blood leukocytes using the standard salting out procedure. The exons of the* NIPBL* gene and their splice junctions were amplified by PCR. The PCR products were purified with USB ExoSAP-IT PCR Product Cleanup (Affymetrix) following the manufacturer's instructions and subsequently sequenced using an ADN 3130 Genetic Analyzer (Applied Biosystems).

*NIPBL* cDNA was numbered according to the* NIPBL* isoform 1 (GenBank accession number NM_000642). The mutation nomenclature was designated following the instructions from the Human Genome Variation Society (http://www.hgvs.org/).

### 2.3. RNA Extraction and Identification of Splice Transcripts by RT-PCR

Total RNA from blood leukocytes of patients and controls was extracted using the PAXgene Blood RNA Kit (PreAnalytiX) according the manufacturer's instructions. Single-stranded cDNAs were synthesized from 500 ng of RNA using the First-Strand Synthesis Kit (Fermentas) with random hexamers.

Specific PCRs were performed for each patient. For patient 1, exons 27–32 were amplified with primers sF27 (5′-GGCCGTTTGCCCAGAGCTTTG-3′) and sR32 (5′-AAACCAGTCATATCCAGTATC-3′). For patient 2, exons 35–38 were amplified with primers sF35 (5′-CATCATCAAATATGGCATGAC-3′) and sR38 (5′-CTAGACCAATGATAGCTTTTG-3′). Each reaction contained 2 *μ*L of cDNA in a 20 *μ*L mixture. Products obtained were analyzed by electrophoresis on a 2% agarose gel. Bands were excised and purified with QIAEX Gel Extraction Kit (QIAGEN), and their identity was confirmed by sequencing.

### 2.4. Minigene Construction and Site-Directed Mutagenesis

Minigene constructs were generated using vector pSPL3 (Exon Trapping System, Gibco, BRL, Carlsbad, CA, kindly provided by Dr. B. Pérez). For patient 1, exon 28 and its intronic flanking regions were amplified by PCR using the primers NM28F (5′-GGCTACAGGTTCTGCAAATG-3′) and NM28R (5′-CCCATGTGGTTCCTATCATC). For patient 2, exon 37 and its intronic flanking regions were amplified using the primers NM37F (5′-ATTACCTGAGGTCGGGAGTTC-3′) and NM37R (5′-GCTGTTGATGTCAACAGTGTGC-3′). The same amplifications were carried out on the genomic DNA from a healthy control.

The fragments were inserted into the pCR-2.1-TOPO vector (Invitrogen Corporation) following the manufacturer's protocol. The inserts were excised with* Eco*RI (Fermentas) and subsequently inserted into pSPL3. Ligation was performed at 25°C for 5 min, using T4 DNA ligase (Invitrogen Corporation).

Mutations introduced on pSPL3-WT were performed with QuikChange Site-Directed Mutagenesis Kit (Stratagene, La Jolla, CA, USA). On pSPL3-28-WT, we disrupted the consensus branch point sequence maintaining the original A at position c.5329-15 (YYRAY→RRYAR) to generate the mutant BP. On pSPL3-37-WT, the pyrimidines deleted in patient 2 were turned into purines to disrupt the polypyrimidine tract of exon 37, generating the mutant PUR.

Plasmids were isolated using the GeneJET Plasmid Miniprep Kit (Fermentas) according to manufacturer's instructions and checked by sequencing.

### 2.5. Minigene Transfection and* In Vitro* Splicing Analysis

HepG2 cells were transfected with 1500 ng of each minigene using JetPEI reagent (Qbiogene Inc., Irvine, CA, USA) following the manufacturer's instructions. Cells were harvested at 48 hours after transfection and RNA was extracted using RNeasy Protect Mini Kit (Qiagen). RT-PCR transcription was carried out with 1 *μ*g of RNA using the First-Strand Synthesis Kit (Fermentas). PCRs were carried out on 5 *μ*L of cDNA using the pSPL3-specific primers SD6 and SA2. The amplified products were analyzed by electrophoresis and sequenced. Each experiment was performed in triplicate.

### 2.6. Real-Time Quantitative PCR

Total* NIPBL* expression was measured in both patients using primers NIPBL35-36F (5′-GGCATGACTGTAGTGCAAC-3′) and NIPBL 36R (5′-ATTGAAACAAGCCCACACAA-3′). Real-time quantitative PCR was performed in an ABI Prism 7000 sequence detector system (Applied Biosystems). We used 1xSYBR Green PCR Mastermix, 25 ng cDNA (total RNA equivalent), and 100 nM primers for each reaction mix. The amplification conditions were 95°C for 10 minutes, followed by 40 cycles of 95°C for 15 seconds and 60°C for 1 minute. Melting curve analysis showed a specific amplification. The same reactions were performed on a pool of four control cDNAs as a reference. All the amplifications were performed in triplicate.

We used standard curves to quantify the expression of* NIPBL*, as well as* GAPDH* for normalization. To create the standard curves,* NIPBL* and* GAPDH* cDNAs were inserted into the pCR-2.1-TOPO vector according to the manufacturer's protocol. Plasmids were quantified by spectrophotometric analysis at 260 nm, and standard curves were based on a 10-fold serial dilution of the different cloned genes. The Ct (cycle threshold) values of the samples were interpolated to the corresponding standard curve. The expression of* NIPBL* was normalized to the expression of* GAPDH* in the same sample. *P* values were calculated using Student's *t*-test.

## 3. Results

### 3.1. Clinical Report

**Figure 1:**
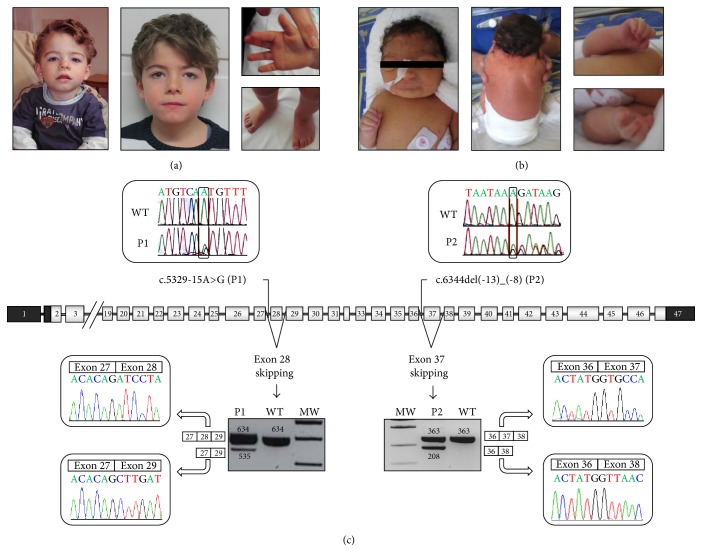
Overview of the phenotype and molecular findings of two patients with CdLS. (a) Phenotype of patient 1. (b) Phenotype of patient 2. (c) Localization of the mutations on* NIPBL* gene and their consequences upon transcript processing. Boxes mean exons and lines mean introns. Dark boxes represent the nontranslated region. The localization of each mutation and chromatograms on genomic DNA are shown above the gene. Agarose gel of the cDNA PCR products in each patient' pictures and chromatograms are shown underneath the gene. Patient 1 yielded normal product of 634 bp and an aberrant fragment of 535 bp corresponding to exon 28 skipping. Patient 2 showed the normal product of 363 bp and an additional band of 208 bp corresponding to exon 37 skipping (MW: molecular weight, WT: wild type, P1: patient 1, and P2: patient 2).

Patient 1 (P1) is an 8-year-old boy who was born after an uneventful pregnancy at 41 weeks of gestation. Birth weight was 3,260 g (−0.8 SD), length 50 cm (−1.5 SD), and head circumference (HC) 34 cm (−1.6 SD). The craniofacial features included arched eyebrows, long eyelashes, hypertelorism, depressed nasal bridge, long philtrum, and thin upper lip. He had small hands with bilateral single palmar crease and short 1st metacarpal (Figure 1(a)). Physical exploration also demonstrated shawl scrotum, micropenis, and hypospadias. Brain Evoked Auditory Response and abdominal and cardiac ultrasounds were normal. At the age of 7 years, his weight was 19 kg (median), his height was 112 cm (−2.2 SD), and his HC was 47 cm (−3.8 SD). Developmental milestones were delayed. More clinical information is provided in Table 1.

Patient 2 (P2) is a 7-year-old girl, who was born at 36 weeks of gestation. Birth weight was 2,130 g (−1.2 SD), length 43 cm (−1.2 SD), and head circumference (HC) 30 cm (−2.2 SD). During the exploration in the newborn period she showed the following features: brachycephaly, arched eyebrows with synophrys, long eyelashes, ptosis, depressed nasal bridge with anteverted nostrils, long and smooth philtrum, thin upper lip, low anterior and posterior hairline with a webbed neck, generalized hirsutism, and* cutis marmorata*. She had small hands with brachymesophalangy V and restriction of elbow movements (Figure 1(b)). During the first months of life, she had a feeding and swallowing disorder, which currently is under treatment, and she has underwent surgery for treatment of gastroesophageal reflux. At the age of one year, her weight was 4630 g (−1.75 SD), height 63 cm (−4.8 SD), and HC 39 cm (−4.8 SD). More clinical information is shown in Table 1.

### 3.2. DNA and RNA Analysis

Sequence analysis of the genomic DNA yielded two novel intronic mutations in the* NIPBL* gene. In patient 1 a transition (c.5329-15A>G in intron 27) and in patient 2 a deletion (c.6344del(-13)_(-8) in intron 36) were identified (Figure 1(c)). Sequencing analysis of the parents confirmed the* de novo* status for both mutations.

RNA analysis could prove aberrant splicing in both patients. In patient 1, an aberrant transcript of 535 bp beside the expected wild type PCR product of 634 bp was observed. Sequencing analysis of the aberrantly spliced product revealed a loss of a 99 bp fragment representing the entire exon 28 region (Figure 1(c)). In patient 2 the expected wild type product of 363 bp plus an additional product of 208 bp was detected. Subsequent sequencing analysis showed a deletion of 155 bp representing exon 37 of the* NIPBL* gene (Figure 1(c)).

### 3.3. Minigene Analysis of the Mutation c.6344del(-13)_(-8)

**Figure 2:**
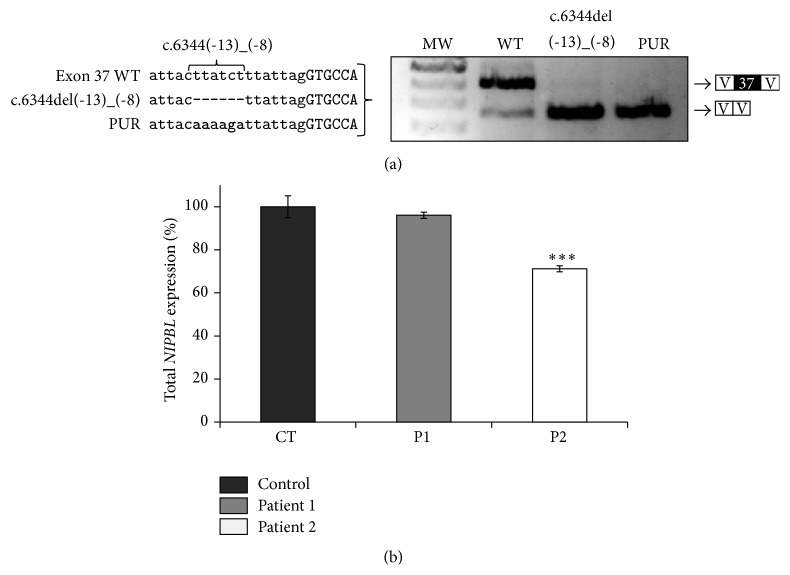
Functional analyses of the intronic mutations c.5329-15A>G and c.6344del(-13)_(-8). (a) Minigene constructs were generated by site-directed mutagenesis. Deletion of c.6344del(-13)_(-8) causes an exon skipping that was also found by a transversion of the five deleted purines into pyrimidines (PUR). (b) Quantitative real-time PCR analysis of total* NIPBL* mRNA from leukocytes of patients with CdLS and controls.* NIPBL* transcript levels were normalized by* GAPDH *expression. The values presented are the medians of triplicate determinations ± SD. The mean ratio of total* NIPBL* mRNA in controls was assigned as 100% (CT: controls, P1: patient 1, P2: patient 2, and *∗∗∗*: *P* < 0.001 versus control).

Transfection using the exon 37 WT construct generated two fragments, the main one of 423 bp containing exon 37 and another of 268 bp without this exon (Figure 2(a)). The presence of the mutation c.6344del(-13)_(-8) leads to a rise of the transcript with exon 37 deletion while the normal transcript disappeared (Figure 2(a)). The disruption of the polypyrimidine tract of exon 37 (PUR) produced a similar effect to mutation c.6344del(-13)_(-8) (Figure 2(a)).

### 3.4. Quantification of* NIPBL* Expression in Patients

Total* NIPBL* mRNA levels were assessed by quantitative real-time PCR using oligonucleotides that recognize* NIPBL* sequences not affected by aberrant or alterative splicing. The mean level of total* NIPBL* expression measured in four control cDNAs (CT) was assigned as 100% (SD = 5.1%), and the mean* NIPBL* expression in each patient was referred as a percentage to the control.

Whereas no distinct changes on* NIPBL* mRNA levels were observed in patient 1 (P1 = 96%, SD = 1.4%) a decrease of ~30% on total NIPBL* mRNA* levels was measured in patient 2 (P2 = 71%, SD = 1.4%, *P* < 0.001) (Figure 2(b)).

## 4. Discussion

Here, we report and characterize for the first time two novel* de novo* CdLS-causing mutations within intronic regions of* NIPBL* that do not affect the conserved splice-donor or acceptor site but result in aberrant mRNA splicing.

In patient 1 (P1), the A>G transition located at c.5329-15 within intron 27 of* NIPBL* results in the skipping of the adjacent exon (Figure 1(c)). This adenine (c.5329-15) lies within a predicted branch point sequence YNYYR$\underline{A}$Y (TGTCA$\underline{A}$T) [16–18], which is in close proximity to the splice-acceptor site and therefore might be involved in the formation of the lariat structure during the splicing process. This kind of mutation is often missed within molecular diagnostics because there is no explicit consensus sequence and branch points can be located at variable distances from the acceptor site [19, 20]. However, the position of this change, the surrounding nucleotides matching the consensus sequence, and A>G transition of the “lariat adenine” is the most frequent mutation affecting proper lariat formation [21], strongly suggesting the disruption of a branch point by this mutation. In our minigene model, exon 28 WT showed a big trend to skip, thus hindering subsequent analyses, maybe due to the lack of some splicing regulatory sequences in the model which are present* in vivo*. Nevertheless, a change in the splicing pattern corresponding to an increase of exon skipping was observed in the presence of the mutation c.5329-15A>G as well as with the disruption of the branch point sequence (data not shown).

The six-nucleotide deletion within intron 36 in patient 2 (P2), c.6344del(-13)_(-8), causes skipping of exon 37 during the splicing process (Figure 1(c)). The deletion disrupts a polypyrimidine stretch located adjacent to the acceptor sequence. Since these stretches are known to play a fundamental role in the splicing process, the deletion of six base pairs, five of which are pyrimidines, within this functional region, might explain the skipping of the exon [22]. Despite the fact that a specific consensus sequence has not yet been defined, it has been reported that a minimum tract is required, and both its length and its composition contribute to splicing efficiency [23, 24]. Although the most common mutations are transversions of pyrimidines into purines [15], this mutation would affect the length of the sequence. The minigene carrying the deletion of c.6344del(-13)_(-8) could further confirm these findings. Furthermore, a transversion of the five deleted pyrimidines into purines (PUR) also results in a skipping of exon 37 which strongly supports the functional relevance of this pyrimidine stretch for proper* NIPBL* splicing (Figure 2(a)).

In general, frameshift mutations in* NIPBL* encoding a truncated nonfunctional protein result in a reduced level of functional NIPBL and are mostly associated with a severe CdLS phenotype due to the genetic model of haploinsufficiency. Missense mutations as well as small in-frame deletions or insertions that preserve the reading frame of the transcript are mostly detected in patients with more moderate phenotypes [4, 25]. The phenotype of the patients with mutations affecting* NIPBL* splicing seems to be in agreement with these observations and are associated with the consequences for the NIPBL protein encoded by the aberrantly spliced transcript [11].

The mutation c.5329-15A>G results in an aberrantly spliced* NIPBL* transcript that excludes a 99 bp fragment representing exon 28 but it preserves the protein reading frame and is supposed to result in a slightly shortened NIPBL protein. This exon encodes parts of HEAT-repeat domain of NIPBL. Although the precise function is still unknown, protein-protein interactions were mapped within the HEAT-repeat domain and there is a clear enrichment of missense mutations affecting this part of the protein [26]. However, at least a partial preservation of the NIPBL function might be postulated, supported by the fact that numerous missense mutations distributed over almost entire protein-coding region of* NIPBL* result in similar CdLS phenotypes [25]. Furthermore, quantitative analyses exclude a significant reduction of the total* NIPBL* transcript levels in patient 1 compared to unaffected controls (Figure 2(b)). This is in contrast to patient 2, who shows a significant reduction of total* NIPBL* mRNA levels down to 71% of those observed in controls (*P* < 0.001) (Figure 2(b)). We could show that the deletion of c.6344del(-13)_(-8) results in an aberrantly spliced transcript that lacks exon 37 which causes a shift in the reading frame. The observed decrease of the* NIPBL* transcript level down to 70% might be explained by nonsense-mediated mRNA decay of the aberrant transcript which would be in agreement with the proposed model of haploinsufficiency [27–30].

The two patients described here meet the general CdLS. It is of note that they present with different phenotypical features. Patient 1 shows a rather mild phenotype without intrauterine growth restriction and a slight postnatal retardation of height and weight. However he shows microcephaly. He has feeding problems without GERD and minor limb anomalies and his developmental delay is mild (Table 1). By contrast, patient 2 shows a more severe phenotype with evident craniofacial features even in the neonatal period, limb abnormalities, profound growth, and psychomotor retardation as well as severe microcephaly. In addition, she suffered from severe GERD (Table 1). The association of frameshift aberrant transcripts with severe CdLS phenotypes has been previously suggested [11]. This work provides experimental support to this hypothesis, since clinical findings are in agreement with our molecular analyses: a general reduction of* NIPBL* mRNA levels and a transcript that contains a frameshift or encodes a truncated protein result in a more severe phenotype than alteration of protein function/structure preserving the reading frame and totally* NIPBL* levels [4, 25].

Furthermore, we could give functional evidence that intronic variants which do not necessarily affect splice-donor or acceptor site result in aberrantly spliced* NIPBL* transcripts and are of pathological relevance for CdLS. Although recent findings could reveal* de novo* mutations in the* ANKRD11* gene in patients with the clinical diagnosis of CdLS, the genetic causes in about 30% of patients with CdLS are still unknown [31, 32]. Whether unknown mutations affecting* NIPBL* splicing within the often large intronic regions that are not totally covered by current sequencing analyses might result in the CdLS phenotype can only be speculated. Thus, intronic variants identified by sequencing analysis in CdLS diagnostics should carefully be examined before excluding them as nonrelevant to disease.

## 5. Conclusions

We report on the functional characterization of two intronic* NIPBL* mutations in two patients with CdLS that do not affect a conserved splice-donor or acceptor site. While c.5329-15A>G may disrupt a branch point in exon 27, c.6344del(-13)_(-8) would affect a polypyrimidine stretch in exon 37. The deletion of c.6344del(-13)_(-8) generates a frameshift aberrant transcript resulting in* NIPBL* haploinsufficiency, consistent with the severe phenotype of this patient. These findings highlight the fact that intronic variants should carefully be examined before excluding them as nonrelevant to CdLS.

## Figures and Tables

**Table 1:** Clinical features of two patients with CdLS.

	Patient 1	Patient 2
Mutation	c.5329-15A>G	c.6344(−13)_(−8)del
Predicted effect on protein	p.(Ile1777_Arg1809del)	p.(Gly2115Valfs*∗*11)
Clinical severity	Mild	Severe
Gender	Male	Female
Anthropometric data (newborn)		
Gestational age	41 weeks	36 weeks
Birth weight	3260 g (−0.55 SD)	2130 g (−1.01 SD)
Birth length	50 cm (−0.46 SD)	43 cm (−1.73 SD)
Birth OFC	34 cm (−1.02 SD)	30 cm (−1.71 SD)
Intrauterine growth restriction	−	+
Anthropometric data (last evaluation)		
Age at evaluation	7 years	1 year
Weight at evaluation	19 kg (−1.31 SD)	4630 g (−4.74 SD)
Length at evaluation	112 cm (−2.14 SD)	63 cm (−4.61 SD)
OFC at evaluation	47 cm (−4.07 SD)	39 cm (−6.05 SD)
Postnatal growth retardation	+	+
Limb defects	Small hands, single palmar crease, short 1st metacarpal	Small hands, brachymesophalangy V, restriction of the elbow movements
Developmental delay	+ Speech delay	+ No speech Learned walking at 3.5 years
Intellectual disability	+	+
Microcephaly	+	+
Behaviour impairment	−	+
GERD	−	+ Floppy Nissen fundoplication
Feeding and swallowing disorders	+	+ No chewing Stomach tube until 5 years
Hirsutism	−	+
Cutis marmorata	−	+
GU anomalies	Shawl scrotum, micropenis hypospadias	NA
Others		Recurrent infections Nuchal cystic hygroma/nuchal edema

+: present; −: not present; OFC: head circumference; CNS: central nervous system; NA: not available; GERD: gastroesophageal reflux disease; GU: genitourinary; SD: standard deviation.
